# The analysis of TOP2A as a potential target for predicting the prognosis of brain glioma in the TCGA database and in single-center study

**DOI:** 10.1007/s12672-025-04051-4

**Published:** 2025-11-24

**Authors:** Tingting Shen, Lei Sheng, Hua Zhan, Sihan Chen

**Affiliations:** 1 Department of Pathology, the First Affiliated Hospital of Anhui Medical University, Hefei, Anhui China; 2 Ningbo Taikang Hospital, Ningbo, China; 3 Department of Radiation Oncology, the Chest Hospital of Anhui Province, Hefei, China

**Keywords:** Ganglioglioma, Glioma, TOP2A

## Abstract

**Background:**

Glioma is a neurological tumor with a high degree of malignancy and a poor survival prognosis. The development of bioinformatics-related databases has helped us to further analyze the correlation between genes and tumors. In previous studies, through GSVA enrichment analysis, we obtained 17 core genes (TOP2A, KIF20A, CCNB2, AURKA, KIF11, CDK1, BUB1B, CCNA2, BUB1, CDC20, CDCA8, TPX2, KIF2C, POLA2, POLE2, POLA1 and POLE) that affect the survival prognosis of patients, and since TOP2A order is the highest we found that TOP2A is associated with the clinical features of brain gliomas through a series of bioinformatics databases and statistical analyses. After undergoing bioinformatics analysis, in this study, we further explore the correlation between TOP2A and the clinical samples of real-world glioma patients, so as to analyze the relationship between TOP2A expression and the survival prognosis of patients. It is worth mentioning that only malignant gliomas were designed in this study, and benign gliomas were not included in the scope of this study.

**Materials and methods:**

Downloaded glioma data from TCGA database. COX regression analysis assessed clinical relevance. Experimental verification by immunohistochemistry. GSEA analyzes relevant biological functions.

**Results:**

Focused on the correlation between TOP2A and glioma, and transported 161 glioma tissue wax blocks from the First Affiliated Hospital of Anhui Medical University, analyzed the correlation between gene expression and clinical data in the real world, and confirmed that it was closely related to the survival prognosis of glioma patients.

**Conclusions:**

Through bioinformatics analysis and immunohistochemical experiments combined with real-world patient data, it was confirmed that TOP2A expression was closely related to patient prognosis.

**Supplementary Information:**

The online version contains supplementary material available at 10.1007/s12672-025-04051-4.

## Introduction

Bioinformatics big data mining plays an important role in tumor research. Bioinformatics big data mining refers to the process of analyzing, mining and interpreting biological big data using computer technology and biological knowledge. In tumor research, bioinformatics big data mining can help scientists better understand the occurrence, development and metastasis mechanism of tumors, and provide important basis for early diagnosis, treatment and prognosis evaluation of tumors. For example, by analyzing the gene expression profile in tumor tissue, scientists can discover genes related to tumors, thus providing new targets for tumor treatment and screening new anti-cancer drugs. In previous studies, we analyzed the gene expression profile of glioma in TCGA database and found that the expression of TOP2A was positively correlated with the staging of glioma [1]. In this study, we retrieved 161 glioma tissue specimens from the pathology center of the First Affiliated Hospital of Anhui Medical University to further explore the expression of TOP2A in glioma staging [2]. TOP2A is a type of topoisomerase II alpha that participates in DNA topological structure conversion. It is highly expressed in many malignant tumors and is expected to become a new molecular marker for tumor prognosis. Glioma is a tumor that occurs in the brain or spinal cord. Recent studies have shown that TOP2A is highly expressed in glioma tissue and may be related to the tumor survival prognosis of glioma. In addition, HCMV infection can promote TOP2A high expression after glioma infection, thereby promoting the malignant progression of glioma [3, 4]. In addition, we collected tissue samples from 161 glioma patients in the First Affiliated Hospital of Anhui Medical University. The expression of TOP2A in each tissue specimen was identified by immunohistochemistry experiments. By integrating the clinical data of patients, the results demonstrated a strong correlation between TOP2A expression and patient survival prognosis. The survival prognosis of patients with high TOP2A expression was significantly poorer than that of the low-expression group, and TOP2A expression was markedly higher in WHO grade III-IV patients compared to those with WHO grade I-II.

## Method

### Data sources

Download relevant gene expression data (529) for glioma from the TCGA database, as well as clinical information. The clinical information and pathological wax block specimens of 161 glioma patients were retrieved from the database of the First Affiliated Hospital of Anhui Medical University.

### Survival analysis

Through log-rank analysis, and plot Kaplan-Meier curve. *P* < 0.05 was considered statistically significant, and the ROC (receiver operating characteristic curve) model was used to analyze its accuracy.

### Clinical correlation analysis

Clinically relevant heat maps are used to show the correlation of clinical features with TOP2A expression groupings (COX analysis) and Nomogram diagrams are used to calculate direct data on each clinical feature and gene expression (COX analysis). Finally, clinical correlation analysis was used to obtain a forest plot of the correlation between clinical data and TOP2A expression.

### Gene co-expression analysis

Through gene expression correlation analysis, the genes that can form an expression trend with TOP2A were obtained, and the circos plot and heat map were plotted, in the circos plot with positive correlation indicated by red and negative correlation represented by green. In the heat map, positive correlations are represented by red and negative correlations by blue.

### Biological functional analysis

Search the biological database for the Gene Set Enrichment Analysis (GSEA) of genes associated with the trend of TOP2A expression, including: Molecular Function; Cellular Component; Biological Process.

### Immunohistochemistry

Staining method: EnVision two-step staining method. Thickness of wax block: 3 μm. Antibody: diluted 1/200, rabbit monoclonal, clone P11388 (Affinity Biosciences Cat#AF0793, RRID: AB_2834111). Scoring criteria: 0 for negative staining, 1 for weak staining, 2 for moderate staining, and 3 for strong staining. Stained areas were stratified as follows: <5% scored 0, 6–25% scored 1, 26–50% scored 2, 51–75% scored 3, and > 75% scored 4. The final staining score for each sample was scored by multiplying the percentage score by the staining intensity. A value ≥ 6 was defined as overexpression.

### Statistical analysis

The correlation between TOP2A expression and clinicopathologic features is that the variable is assessed by chi-square test (categorical variable) or two-sample t-test (continuous). All statistical tests were bilateral. All analyses in this paper concluded that *P* < 0.05 was statistically significant.

### Human ethics

The human tissue specimens used in this study were approved by the Ethics Committee of the First Affiliated Hospital of Anhui Medical University and the Ethics Committee of Ningbo Taikang Hospital (Approval No.: Quick-PJ 2023-08-44). All experimental procedures involving human subjects strictly adhered to the ethical standards of the institutional research committees, the Declaration of Helsinki (1964), and its subsequent amendments. Written informed consent for the collection and research use of tissue samples was obtained by the Department of Pathology at the First Affiliated Hospital of Anhui Medical University. Informed consent was obtained from all subjects and/or their legal guardian(s).

## Result

### Trends in the expression of TOP2A in gliomas

**Fig. 1:**
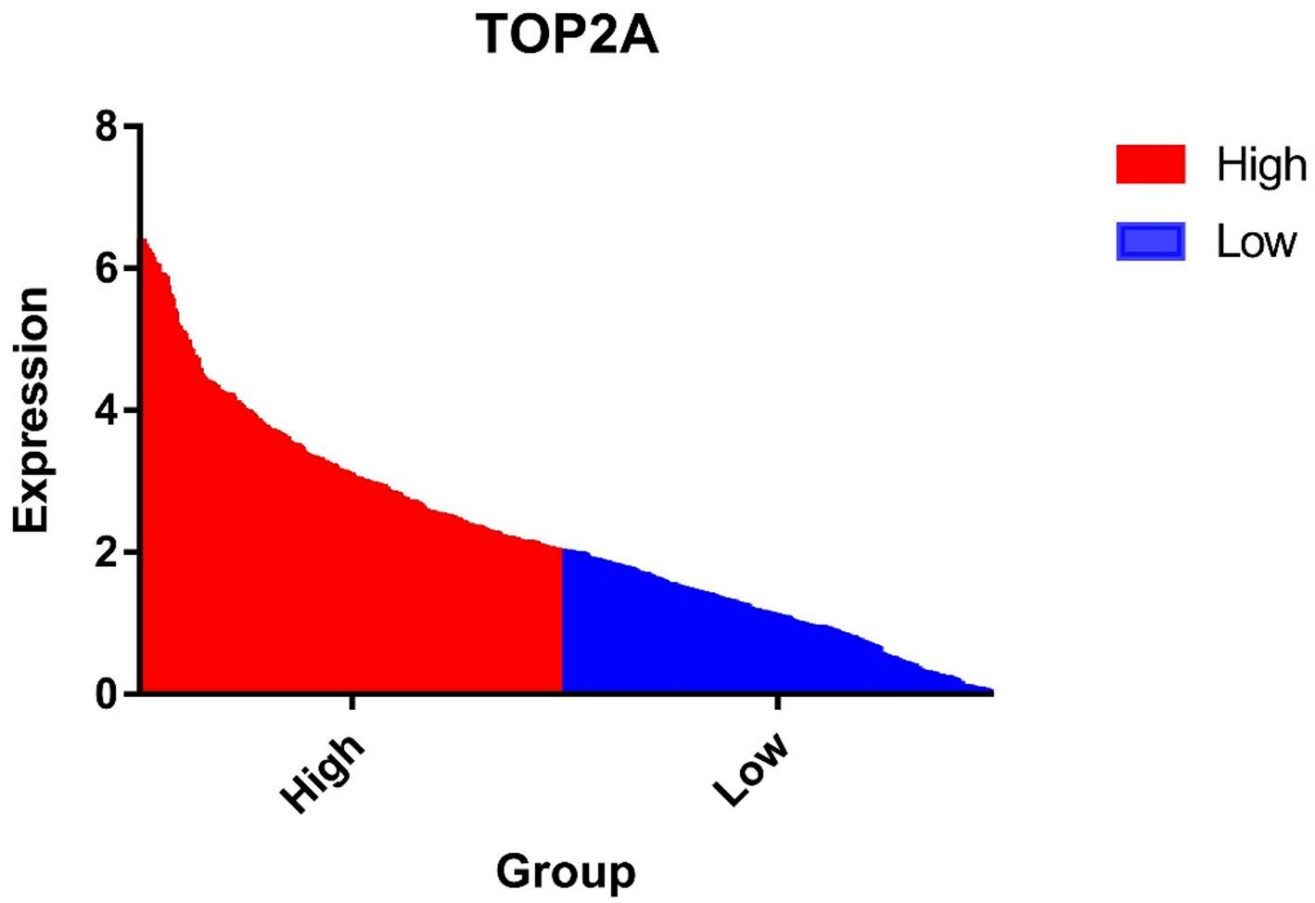
Distribution trend of TOP2A high expression group and low expression group

According to the TOP2A expression of brain gliomas obtained in the TCGA database, we divided them into high-expression groups and low-expression groups (according to median) to obtain their gene trend maps (Fig. 1).

### The expression of TOP2A correlates with the clinical prognosis of glioma

**Fig. 2:**
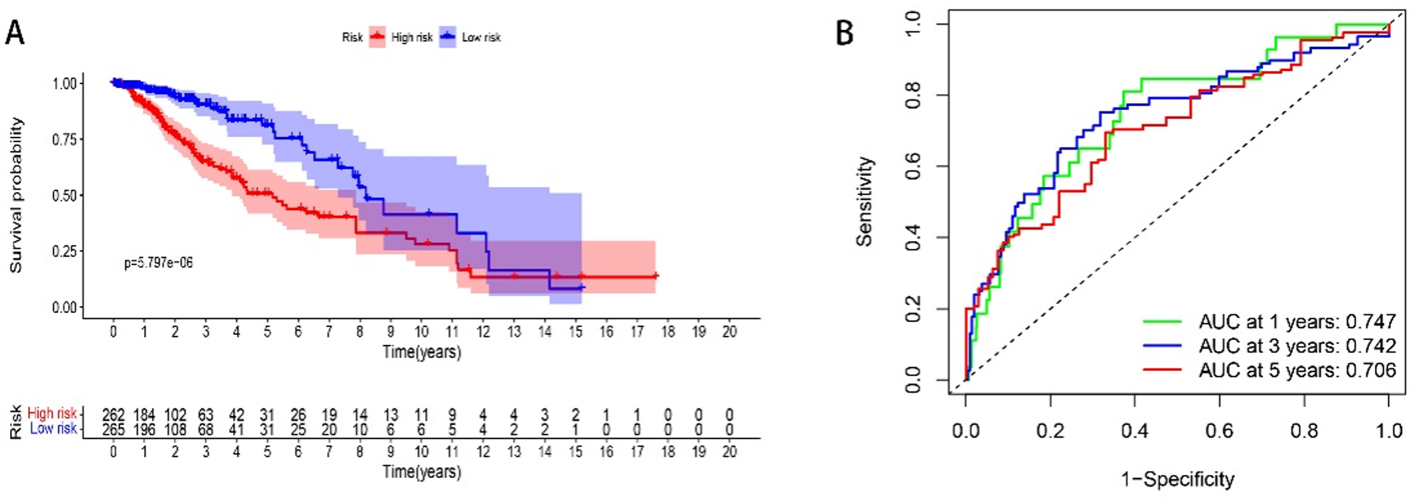
Survival prognostic model. **A** K-M curve, the survival rate of TOP2A high standard group was significantly worse than that of low expression group. **B** The AUC value of the ROC model was greater than 0.7, indicating that the accuracy of the prognostic model was good (*P* < 0.05 is considered statistically significant)

Correlation analysis (K-M survival curve) was performed in connection with the expression of TOP2A in glioma with clinical prognosis, and the results showed that the survival prognosis of the TOP2A high expression group was significantly worse than that of the low expression group. The ROC model shows that the AUC index is greater than 0.7, indicating that the model accuracy is good (Fig. 2).

### Gene expression and clinical correlation analysis

**Fig. 3:**
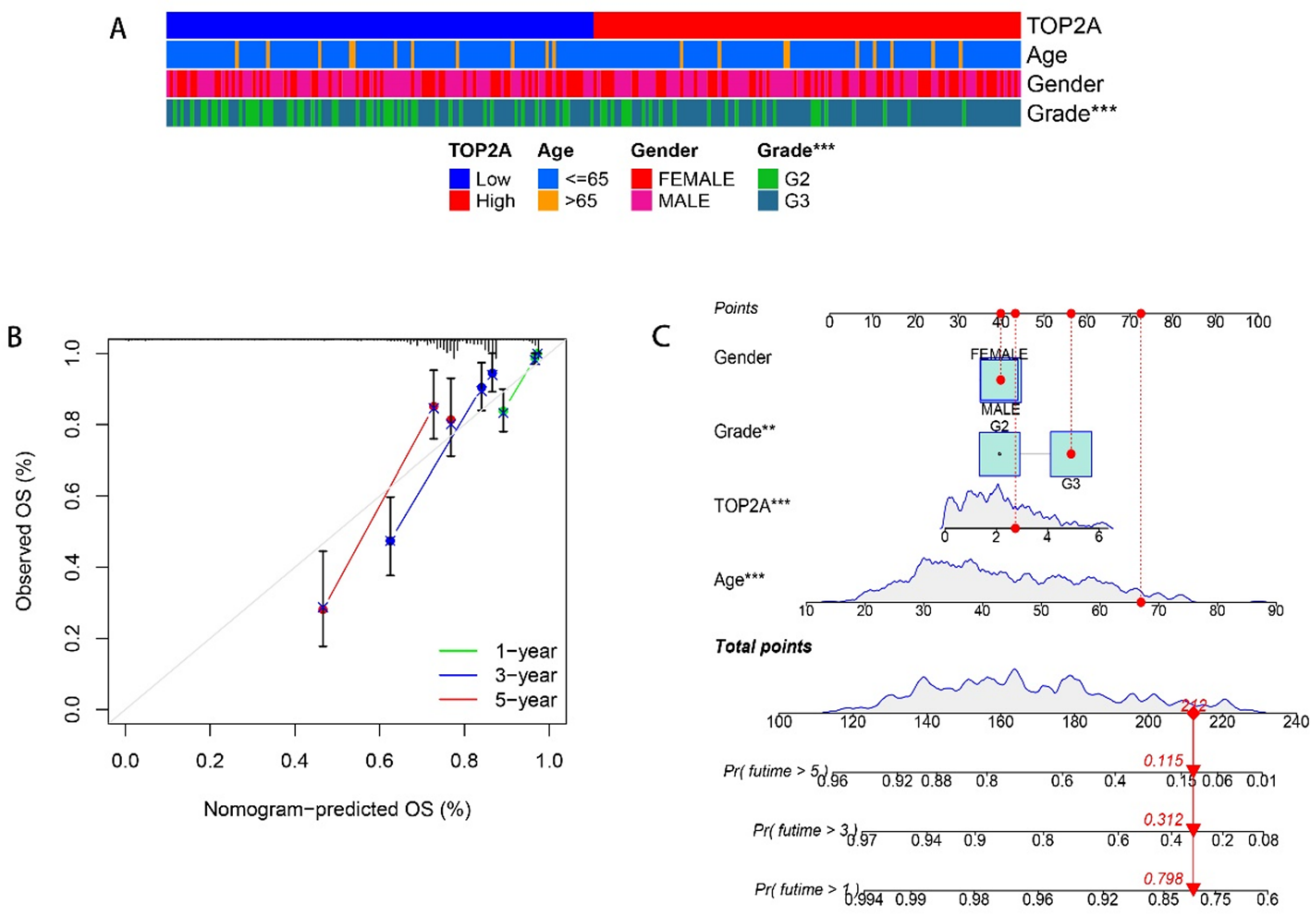
Nomogram analysis. **A** Heat map of clinical factors associated with gene expression quantities. **B** Nomogram-predicted OS, 1, 3, 5 years of forecasts. **C** The distribution of scores for each clinical indicator assessing prognosis (*P* < 0.05 is considered statistically significant)

**Fig. 4:**
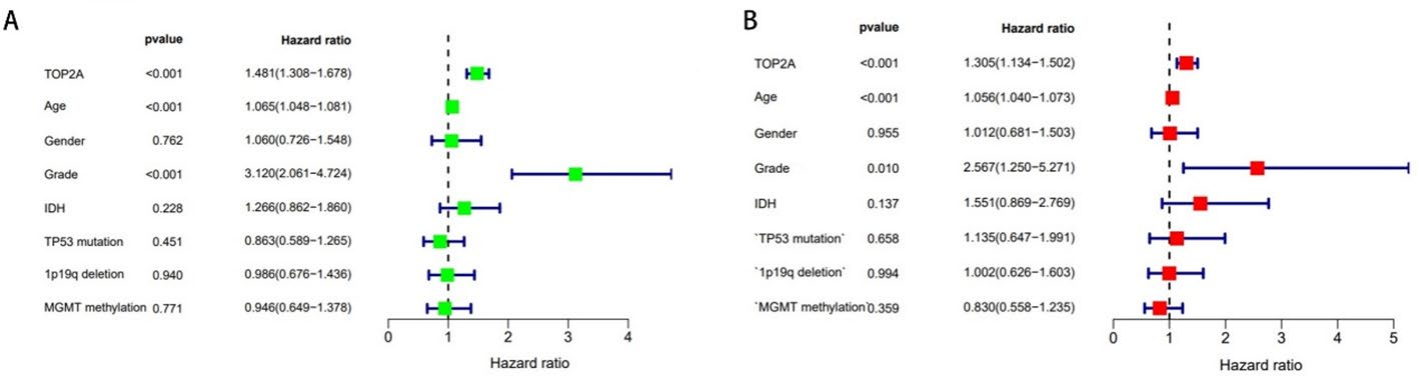
COX clinical correlation analysis. **A** Univariate COX analysis. **B** Multivariate COX analysis (*P* < 0.05 is considered statistically significant)

Glioma has different clinical features than some tumors and does not have TNM stage. Combined with the analysis results of our study, it was found that the grade of glioma was significantly correlated with TOP2A expression. However, Nomogram also suggests that the age of the patient must also be the survival rate of Chengdu imaging patients, which is related to the basic situation of the human body (Fig. 3). Univariate COX regression showed that TOP2A, AGE, GRADE, IDH mutation, and 1p19p deletion are considered clinically relevant to glioma. But multivariate COX regression analysis showed that TOP2A, AGE, and GRADE are considered clinically relevant to glioma (Fig. 4).

### Gene correlation and biological function analysis

**Fig. 5:**
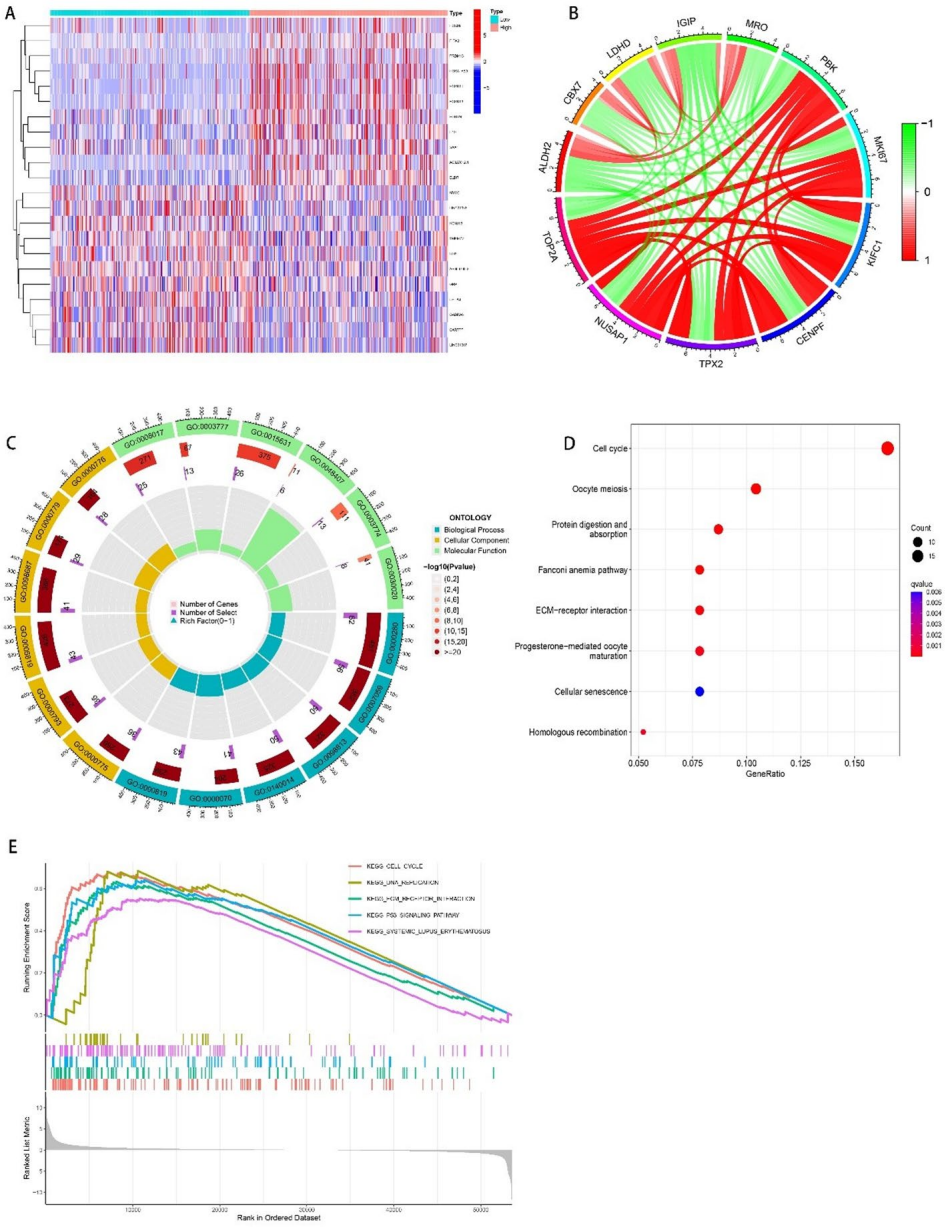
TOP2A-related gene analysis. **A** Heat map of genes with strong correlation with TOP2A, red represents positive correlation and blue represents negative correlation. **B** Correlation circle plot of the above genes, red represents positive correlation and green represents negative correlation. **C** Biological function analysis of the above genes (GO analysis). **D**, **E** Biological function analysis of the above genes (KEGG analysis) (*P* < 0.05 is considered statistically significant)

**Fig. 6:**
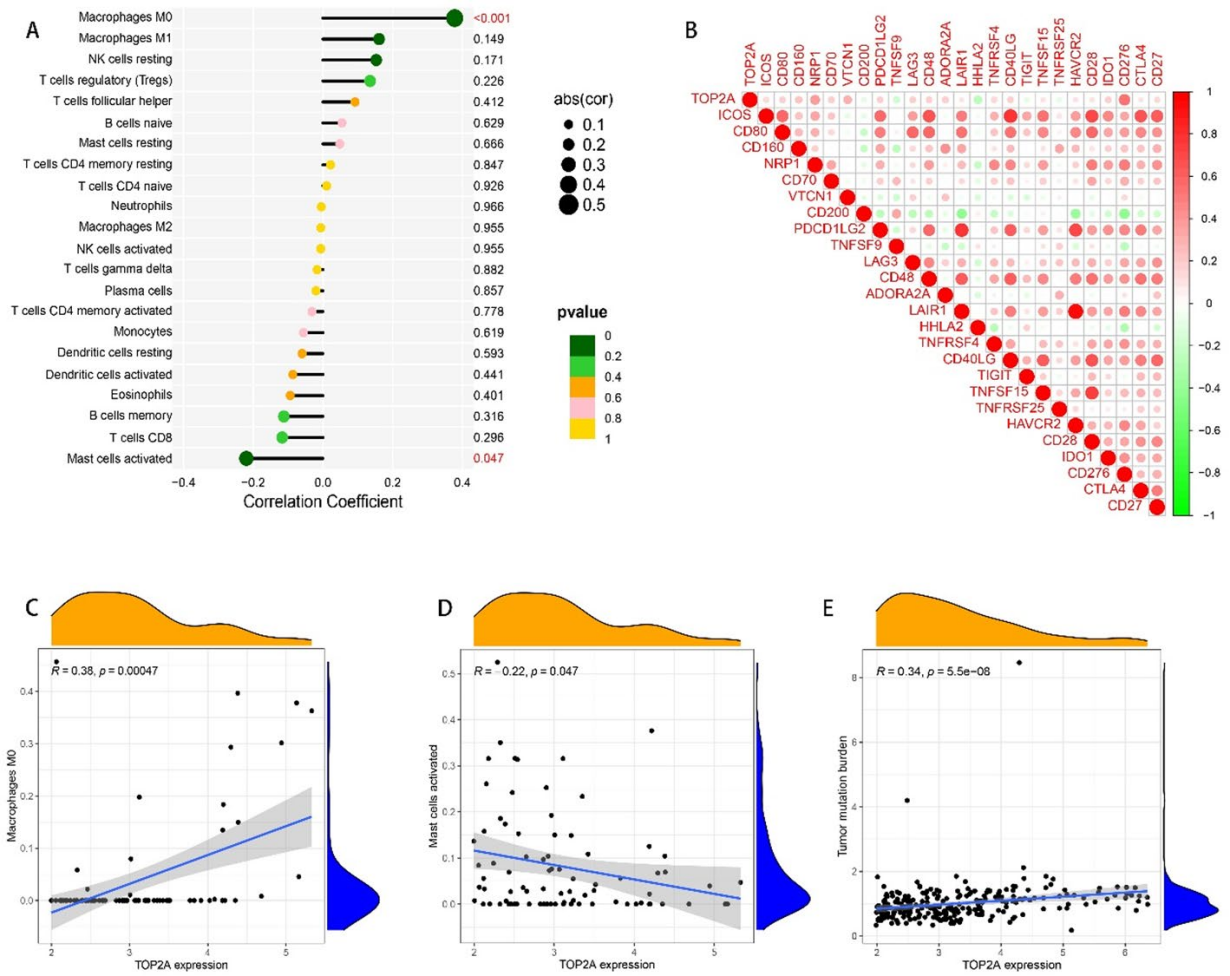
Correlation analysis of TOP2A and immune cells and genes. **A** Correlation analysis between TOP2A and immune cells, Macrophages M0 and TOP2A expression have a certain correlation. **B** The correlation between TOP2A and immune genes was analyzed, and the correlation with CD276 was the strongest. **C** Expression of TOP2A and trend chart of various immune data (*P* < 0.05 is considered statistically significant)

According to the expression trend of TOP2A in glioma, we screened some genes with strong correlation, (Fig. 5A, B), the strongest correlation is CD276 (Fig. 6B)), and their biological function suggested that they were more related to cell cycle and oocyte meiosis (Fig. 5C, D, E). A search for its correlation with immune cells in the CIBERSORT database showed that Mast cells activated decreased with increasing expression of TOP2A (*P* < 0.05). The expression of Macrophages M0 and tumor mutation burden was correlated with the expression of TOP2A (*P* < 0.05). (Fig. 6A, C, D, E).

### Immunohistochemical assessment-correlation analysis of gene expression with clinical data

**Fig. 7:**
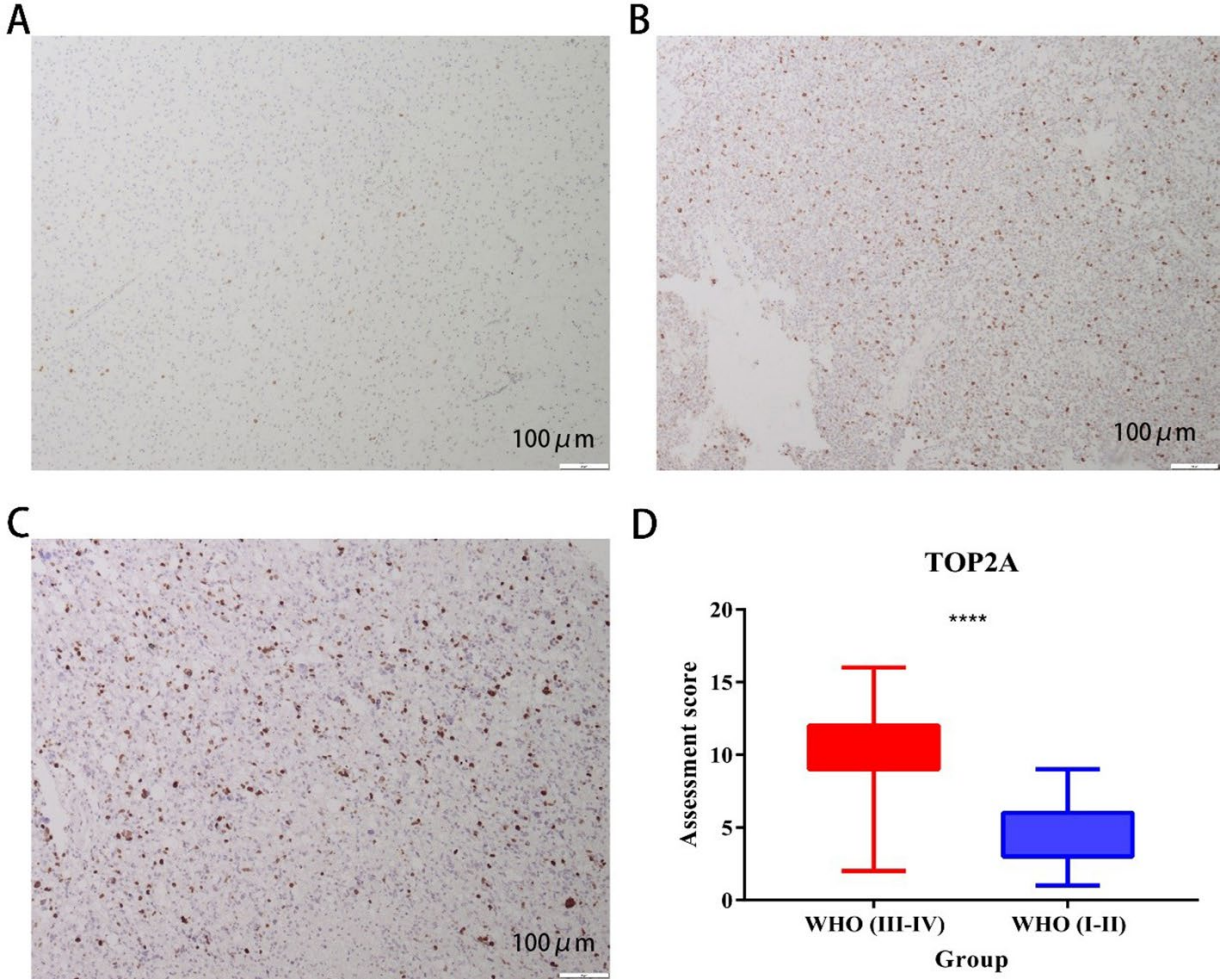
Representative IHC of TOP2A staining intensity expression and box plot expression of TOP2A staining score in glioma. **A** TOP2A staining intensity was weakly positive in glioma. (Magnification, x100; EnVision two-step). **B** TOP2A staining intensity was moderately positive in glioma. (Magnification, x100; EnVision two-step). **C** TOP2A staining intensity was strongly positive in glioma. (Magnification, x100; EnVision two-step). **D** Box plot expression of TOP2A staining score in low-grade glioma and high-grade glioma. (*P* < 0.05 is considered statistically significant)

**Fig. 8:**
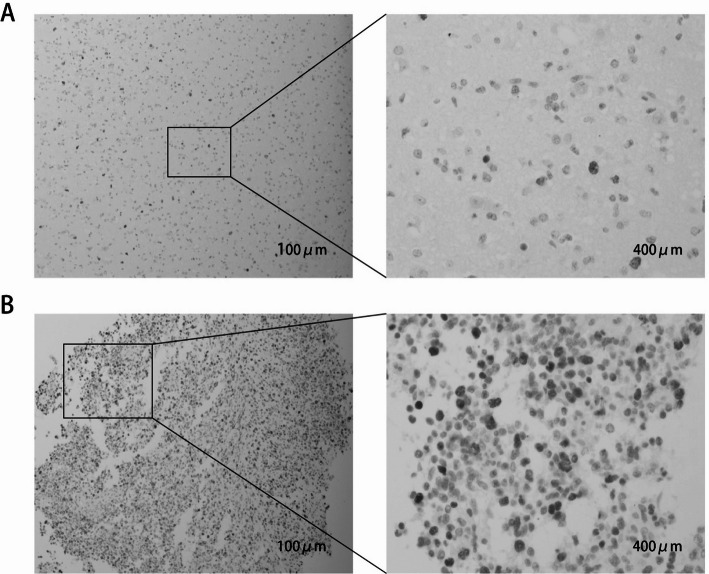
Representative IHC of TOP2A staining intensity expression in glioma (Images were taken by OLYMPUS SC180 microscope.) **A** A low-grade glioma tissue showing low TOP2A protein expression (magnification, x10 left and x40 right). **B** A high-grade glioma tissue showing high TOP2A protein expression (magnification, x10 left and x40 right)

The expression of TOP2A in glioma tissues was evaluated by immunohistochemistry, and the results showed that TOP2A was significantly correlated with the gliomas of varying degrees of malignancy (Because we collect fewer cases with WHO grade I, they will not be discussed in this study.) (*P* < 0.001). K-M survival analysis showed that the survival prognosis of TOP2A high expression group was significantly worse than that of low expression group. The ROC model showed that the AUC values of the 1, 3 and 5-year survival models were above 0.7, indicating that the model accuracy was good. Clinical correlation analysis (COX analysis) showed that TOP2A expression was significantly correlated with patient survival prognosis in both univariate and multivariate clinical correlation analysis (Fig. 7). Representative IHCs are expressed at TOP2A staining intensity in gliomas, and images taken by Olympus SC180 microscopy distinguish immunohistochemistry between high-expression and low-expression groups (Fig. 8).

### Survival analysis with real-world specimens

**Fig. 9:**
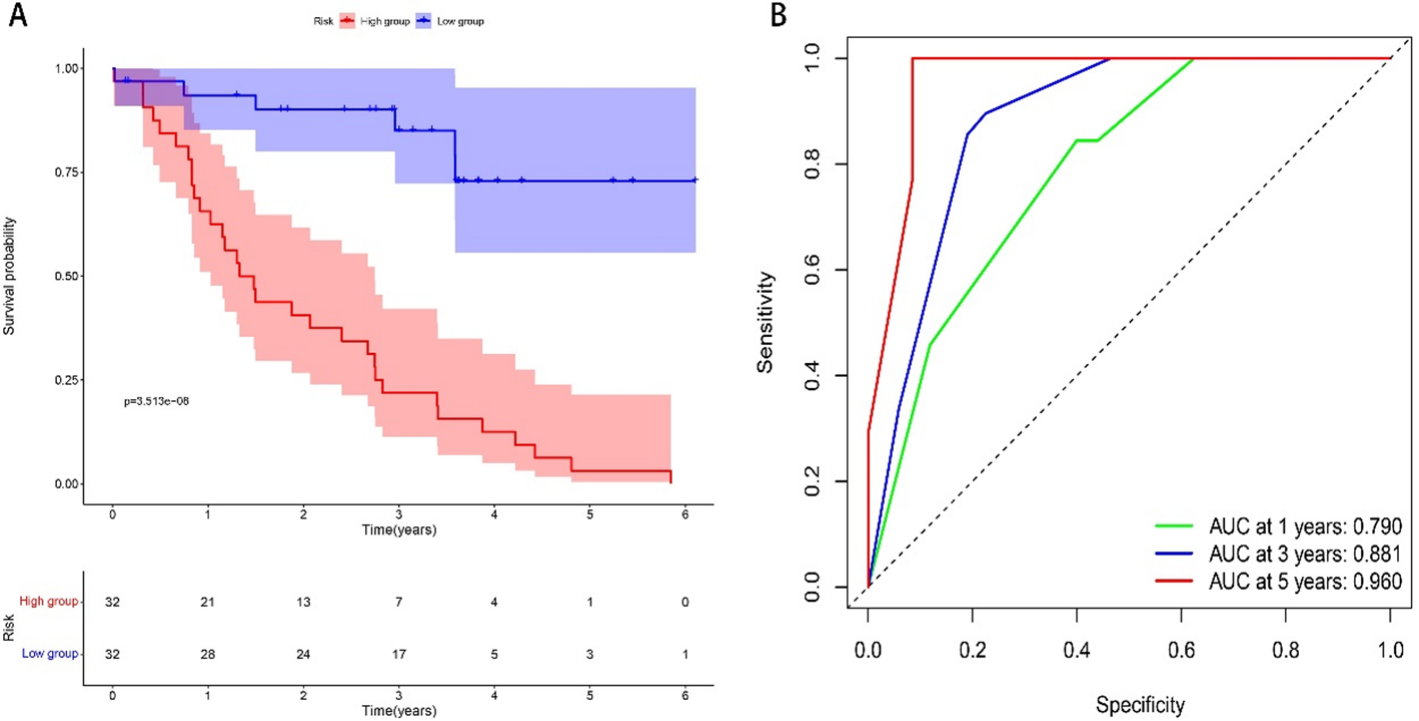
Survival prognostic model in real world clinical samples. **A** K-M curve, the survival rate of TOP2A high standard group was significantly worse than that of low expression group. **B** The AUC value of the ROC model was greater than 0.7, indicating that the accuracy of the prognostic model was good (*P* < 0.05 is considered statistically significant)

Using the survival analysis of clinical samples from the First Affiliated Hospital of Anhui Medical University, the methods were the same as the above survival model, and the results showed that the survival prognosis of the high-expression group was significantly worse than that of the low-expression group, and the AUC index was greater than 0.7, indicating that the accuracy of the model was good (Fig. 9).

### TOP2A protein expression and correlation with clinicopathological characteristics

**Fig. 10:**
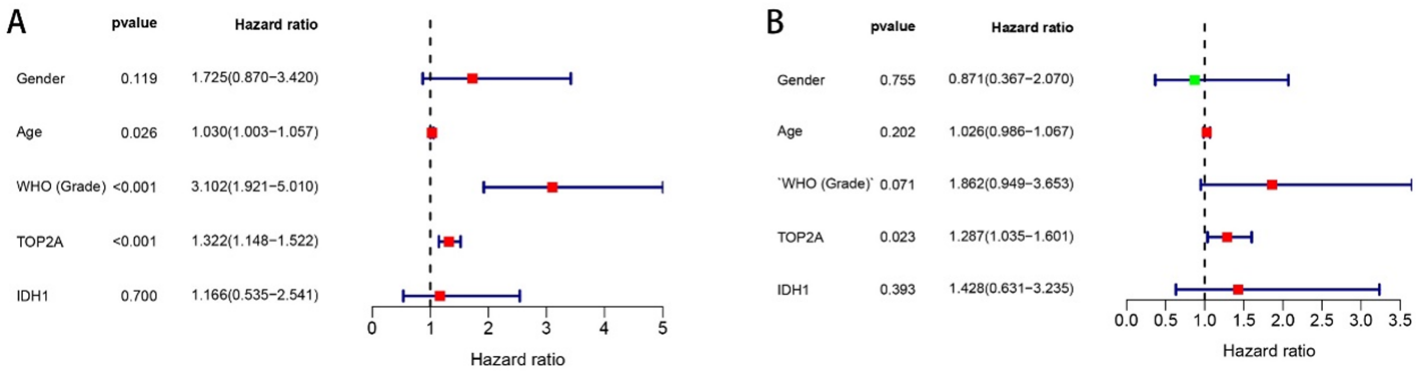
COX regression analysis based on data collected from the First Affiliated Hospital of Anhui Medical University. **A** Univariate COX analysis. **B** Multivariate COX analysis (*P* < 0.05 was considered statistically significant)

The variable positive expression of TOP2A in all 160 glioma tissues was detected by IHC. Representative micrographs showing the staining intensity of TOP2A in glioma tissues are shown in Fig. 8. We conducted COX regression analysis using data collected from the First Affiliated Hospital of Anhui Medical University. Univariate COX regression analysis demonstrated that patient age, tumor WHO grade, and TOP2A expression level were significantly associated with hazard ratio (*P* < 0.05). Multivariate COX regression analysis further confirmed that TOP2A remained significantly correlated with hazard ratio (*P* < 0.05) (Fig. 10). Regarding the clinical content of the analyzed data, we have summarized it in Table 1, with more detailed data uploaded to the supplementary files.

**Table 1:** Distribution of clinical data collected from The First Affiliated Hospital of Anhui Medical University

Characteristic	Total (*n* = 160)	High group (*n* = 74)	Low group (*n* = 86)
*Age at surgery (years)​​*			
< 65	116 (72.5%)	49 (66.2%)	67 (77.9%)
≥ 65	44 (27.5%)	25 (33.8%)	19 (22.1%)
*Gender​*			
Male	105 (65.6%)	58 (78.4%)	47 (54.7%)
Female	55 (34.4%)	16 (21.6%)	39 (45.3%)
*WHO Grade​*			
I-II	44 (27.5%)	2 (2.7%)	42 (48.8%)
III-IV	116 (72.5%)	72 (97.3%)	44 (51.2%)
*IDH1 status*			
Positive	52 (31.7%)	22 (29.7%)	30 (34.9%)
Negative	33 (20.1%)	16 (21.6%)	17 (19.8%)
Not examined	75 (45.7%)	36 (48.6%)	39 (45.3%)

Data are presented as n (%)

## Discussion

Glioma is a very malignant neurological tumor. The annual incidence is about 3–8 per 100,000 population. Glioma often has a poor prognosis because of its high degree of malignancy and the difficulty of complete surgical resection. It is particularly important to find effective gene targets before the development of gliomas to the extent that surgery is difficult to remove, as well as target therapy. With the development of bioinformatics related technologies, I can more efficiently screen out genes that affect the occurrence and development of gliomas through big data analysis [2]. A large number of studies have shown that genes are closely related to the development of tumors. Immunohistochemistry is a commonly used method for verifying gene expression that directly visualizes the distribution and content of target proteins at the tissue or cellular level. Immunohistochemistry has strong specificity, can accurately bind to the target protein, avoid mixed cross-reactions, and its sensitivity is also high, and it has a localization effect (can identify the position of the target protein in the tissue). After we collect the clinical information of the corresponding patients of the organization, we can evaluate the relationship between the expression of the gene and the survival prognosis of the patient by immunohistochemistry combined with relevant statistical analysis.

TOP2A is an enzyme that controls the state of DNA topology, catalyzes double-stranded DNA breaks and promotes gene transcription during mitosis. Abnormal expression of TOP2A is associated with the malignant characteristics of the tumor, for example, the level of expression of TOP2A in small cell lung cancer is closely related to patient prognosis. TOP2A plays a carcinogenic role in a variety of tumor types. These studies show that TOP2A plays an important role in tumors. TOP2A is an enzyme that catalyzes double-stranded DNA breaks and facilitates gene transcription during mitosis. It plays an important role in normal cells, but in some cases, it can cause DNA damage and mutations. Abnormal expression of TOP2A correlates with malignant features of tumors. For example, in small cell lung cancer, the expression level of TOP2A is closely related to patient outcomes. In addition, TOP2A plays a carcinogenic role in a variety of tumor types. The mechanism of action of TOP2A in tumors is not fully understood. However, some studies suggest that it may promote tumor growth and metastasis in a number of ways. For example, in breast cancer, high levels of TOP2A expression are associated with HER2-positive breast cancer and may promote tumor growth by regulating the HER2 signaling pathway. In addition, in colorectal cancer, high levels of TOP2A expression are associated with malignancy and poor prognosis. In conclusion, TOP2A plays an important role in tumors and could be a promising therapeutic target [5–7]. TOP2A is one of the major members of the TOP2 family and is able to catalyze DNA double-strand breaks to induce gene transcription during mitosis. Previous studies have shown that TOP2A is highly expressed in several types of cancers, including breast cancer and uroepithelial carcinoma, and serves as a biomarker for these cancers. It was reported that once the TOP2A expression level decreased, the β-catenin level was also downregulated, indicating that the Wnt/β-catenin signaling pathway was inhibited, suggesting that the reduction of TOP2A level is beneficial to improve the prognosis of tumor patients. In contrast, higher levels of TOP2A imply an active proliferative state of cancer cells, which can enhance tumor aggressiveness and lead to disease progression, resulting in poor prognosis and increased risk of death. In our study, high-grade glioma tissues were observed to express high levels of TOP2A by immunohistochemical staining methods compared to low-grade glioma tissues. In addition, the results of this study showed that TOP2A was expressed at significantly higher levels in male patients than in females. Therefore, high expression of TOP2A is one of the important target genes that promote the development of high-grade glioma and is a potential target for the diagnosis and treatment of high-grade glioma [8, 9].

In this study, we downloaded the gene expression data and clinical data of glioma in TCGA to analyze the direct relationship between TOP2A expression and patient survival prognosis and the correlation with patient clinical information.

Through the expression distribution (Fig. 1), we can find that the expression degree of TOP2A in glioma is different, and there is a certain trend change. Surgery for gliomas is different from some tumors, we cannot remove normal brain tissue, but the largest, safe removal of the tumor, so we cannot perform a control of tumor tissue with normal brain tissue, in the same patient. Therefore, we set the median of TOP2A expression as the grouping boundary, the higher than median set as the high expression group, and the lower than median set as the low expression group. After the grouping is completed, the relevant model can be calculated by combining the clinical data of patients with different groupings.

In order to improve the accuracy of diagnosis and treatment of gliomas, the European Association of Neuro-Oncology (EANO) incorporated molecular markers such as IDH mutation (IDH-mu), chromosome 1p or 19q co-deletion (1p/19q-codel) and MGMT promoter methylation (MGMTp-M) into the diagnostic guidelines for gliomas [10]. Zixi Yang et al. showed that IDH-mu only and the combination of IDH-mu and MGMTp-M with or without 1p/19q-codel carry a favorable prognosis in high-grade gliomas, wihle p53+/Ki-67 + predicts adverse outcomes in these patients. In this context, we incorporated the above data into the COX regression analysis. Univariate COX regression showed that TOP2A, AGE, GRADE, IDH mutation, and 1p19p deletion are considered clinically relevant to glioma. But multivariate COX regression analysis showed that TOP2A, AGE, and GRADE are considered clinically relevant to glioma. Only data that were statistically significant in multivariate cox regression analysis were included in the Nomogram analysis [11]. Nomogram analysis can reasonably analyze the scores of each factor to calculate the impact of each clinical factor on survival prognosis. In this analysis, we showed that TOP2A expression, grade, and Age were all clinical factors that significantly affected the survival prognosis of patients, and TOP2A expression showed significant statistical significance in Nomogram analysis. Through the biological function analysis of GSEA, genes with strong correlation with TOP2A and abnormally expressed to a certain extent in gliomas can be found. They are: MK167, PBK, MRO, IGIP, LDHD, CBX7, ALDH2, NUSAP1, TPX2, CENPF, and KIFC1, and these genes are mainly related to each other through the cell cycle [12].

The MK167 gene is a gene encoding the protein MK167, which plays an important role in cell division and migration. Research on MK167 in gliomas is lacking. The MK167 gene is mutated in some tumors, leading to overexpression or dysfunction of the protein. This may promote the proliferation and invasion of tumor cells, thereby accelerating tumor development and progression. However, several clinical trials are exploring potential therapeutic strategies targeting the MK167 protein or its associated signaling pathway. But some progress has been made in breast cancer [13]. PKB cells have the potential to be used as glioma targets in basic research, but the detailed mechanism needs further study. The research of MRO, IGIP, LDHD, CBX7, ALDH2, NUSAP1, TPX2, CENPF, and KIFC1 in glioma still lacks relevant clinical research evidence, and some theories only stay in cell and animal studies, and further elaboration of the mechanism is still needed.

Finally, we used immunohistochemistry experiments to perform quantitative and qualitative analysis of TOP2A in glioma tissue. In the First Affiliated Hospital of Anhui Medical University, we collected the clinical information of the corresponding patients of the specimens, and finally statistically analyzed the relationship between TOP2A expression and the survival prognosis of patients by disease classification in the real world. The results also support our analysis in bioinformatics. Patients with high TOP2A expression have a poor survival prognosis, and the WHO grade is also higher.

### Limitations

This study does not directly prove the mechanism of the occurrence and development of TOP2A and glioma, but to a certain extent, it reveals their interconnection, and the specific mechanism needs to be further revealed in future studies. Data collected at the First Affiliated Hospital of Anhui Medical University indicates that a substantial proportion of patients did not undergo subsequent immunohistochemical examinations for IDH1 and 1p/19q. One contributing factor may be the comparatively lower economic development and healthcare standards within Anhui Province. This context potentially leads to limited financial resources among patients and/or a degree of skepticism regarding the capabilities of local medical institutions, thereby contributing to the avoidance of further diagnostics. Notably, a subset of patients sought advanced care in cities with superior medical resources, such as Beijing or Shanghai, while others discontinued treatment due to financial constraints (Fig. 10).

## Conclusions

Bioinformatics analysis and immunohistochemistry experiments combined with clinical data show that TOP2A expression is related to patient survival prognosis and pathological grade. However, its specific biological mechanism still needs further study.

## Supplementary Information

Supplementary Material 1.

## Data Availability

Publicly available in a repository: PRO-Seq data were deposited into The Cancer Genome Atlas Program database and are available at the following URL: [https://portal.gdc.cancer.gov/].

## Abbreviations

GSEA: Gene set enrichment analysis
ROC: Receiver operating characteristic
KEGG: Kyoto Encyclopedia of Genes and Genomes
GO: Gene Ontology Analysis
